# Clinical analysis of 23 cases of steroid-associated osteonecrosis of the femoral head with normal initial magnetic resonance imaging presentation

**DOI:** 10.1097/MD.0000000000008834

**Published:** 2017-12-08

**Authors:** Feng-Chao Zhao, Huai-Xia Hu, Xin Zheng, Ding-Wei Cang, Xiaoyun Liu, Jian-Zhi Zhang, Kai-Jin Guo

**Affiliations:** a Department of Orthopedic Surgery, the Affiliated Hospital of Xuzhou Medical University; b Department of Rheumatism, the Second People's Hospital of Lianyungang City, Lianyungang; c Central Laboratory, the Affiliated Hospital of Xuzhou Medical University, Xuzhou, Jiangsu, China.

**Keywords:** corticosteroid, MRI, ONFH, pain

## Abstract

To explore the clinical characteristics of steroid-associated osteonecrosis of the femoral head (ONFH) presenting initially normal magnetic resonance imaging (MRI) results.

This retrospective study examined data from 23 cases that suffered from ONFH but presented a normal image at the first MRI examination after corticosteroid therapy from June 2005 to December 2013. Data on protopathy, age, sex, time of pain onset, MRI examination, and initial diagnosis were collected and analyzed.

Average time from steroid therapy to first MRI examination was 45.7 ± 25.5 days (range, 10–94 days). Average time to final diagnosis was 199.9 ± 165.8 days (range, 32–762 days). Of the 23 cases, 21 cases complained of discomfort and were misdiagnosed because of a normal initial MRI scan. Twelve hips progressed to collapse and 1 hip received lumbar discectomy when got the final diagnosis. Cases with continuous pain (9/21) presented with pain at a later time than those with intermittent pain (12/21), although the continuous pain cases were diagnosed earlier.

MRI performed 2 to 3 months after steroid therapy may present normal images. Another MRI examination is necessary to make a definite diagnosis.

## 1 Introduction

Corticosteroid administration for the treatment of various disorders is one of the main causes of osteonecrosis of the femoral head (ONFH). Average age of people affected by this intractable disease is quite young, at about 38 years old.^[1–3]^ Early detection of ONFH is key to achieving good curative results.^[4–9]^Currently, magnetic resonance imaging (MRI) is universally accepted as the most accurate method for its early diagnosis.^[2,3,10,11]^ However, there is a delay between corticosteroid use and ONFH development^[12,13]^ and even after ONFH has emerged, it may not be detectable on MRI.^[14,15]^ Knowledge of this timeline is essential to explore the mechanism of ONFH, to design interventions to reduce the occurrence of ONFH, and to develop screening plans for high-risk patients.

Unfortunately, to our knowledge, no studies have focused on the false-negative period of steroid-associated ONFH. Therefore, the purpose of this study was to conduct a retrospective analysis of the clinical characteristics of patients who suffered steroid-associated ONFH in the false-negative period.

## 2 Patients and methods

### 2.1 Study design and patients

A retrospective study was conducted with a cohort of 23 patients who had received steroid therapy for different reasons and were confirmed to have ONFH, but presented normal results at their initial MRI examinations. The study took place from June 2005 to December 2013. The ethics committee of our hospital approved the study protocol, and all patients provided written informed consent. Mean age of subjects was 33.2 ± 10.1 years (range, 19–61 years). There were 11 men and 12 women. Fifteen patients suffered bilateral ONFH and 8 suffered unilateral ONFH. Corticosteroid dosages ranged from 367 to 7544 mg, and the median dosage was 2150 mg (prednisone equivalent). Of the 23 patients in the study, 21 underwent initial MRI examinations because they felt discomfort. The other 2 patients received their initial MRI examinations for osteonecrosis screening.

### 2.2 Clinical observations

Data on protopathy, types of corticosteroids taken, dosage of corticosteroids taken (calculated as the prednisone equivalent), time of pain onset, location of initial pain, time of initial MRI, time of final diagnosis, and stage (according to the Association Research Circulation Osseous [ARCO] international staging system proposed in 1993)^[16]^ were collected. Patients were asked about their pain status (continuous or intermittent) at each of the 2 MRI examinations.

### 2.3 MRI protocol and ONFH criteria

Two MRI protocols (coronal T1-weighted imaging and coronal short τ inversion recovery [STIR]) were conducted on 2 machines: aSigna Excite 1.5-T Imager (GE Medical Systems, Milwaukee, WI) and a Philips 1.5-T Imager (Philips Medical Systems Netherlands B.V.). With the Signa machine, coronal T1-weighted sequences (repetition time [TR] 400/echo time [TE] 8.6/Ef) were obtained using a pelvic phased array coil and coronal STIR images were obtained using a TR of 2560 ms and a TE of 108 ms. Images (4-mm thick with 1-mm gaps and a 34 × 34-cm field of view) were obtained using a 256 × 192 matrix with the number of excitations (NEX) set to 4. With the Philips machine, coronal STIR images (TR 2,500/TE 80) and coronal T1-weighted sequences (TR 340/TE 15) were obtained with a 5-mm thickness with 2-mm gaps. A low signal band on the T1-weighted image and a high signal band on the corresponding STIR sequence were deemed as the MRI diagnostic criteria of ONFH.^[16]^

### 2.4 Statistical analysis

Data analysis was carried out in SPSS software (version 12.0, SPSS Inc., Chicago, IL). Age of patients, time of pain onset, time of first MRI examination, time of final diagnosis, and steroid dosage were analyzed by using Student *t* test. The chi-squared test was used to analyze relationships between sex, pain status, pain location, and steroid type. A *P*-value <.05 was considered significant.

## 3 Results

Clinical data for 23 patients, including protopathy, corticosteroid type, corticosteroid dosage, time of pain onset, location of initial pain, time of initial MRI, and time of final diagnosis, are summarized in Table 1. Average time between steroid therapy and first MRI examination was 45.7 ± 25.5 days (range, 10–94 days). Average time between steroid administration and final diagnosis was 199.9 ± 165.8 days (range, 32–762 days). At the second MRI examination, 12 hips had progressed to stage III, 22 hips had progressed to stage II, and 4 hips had progressed to stage I.

**Table 1:**
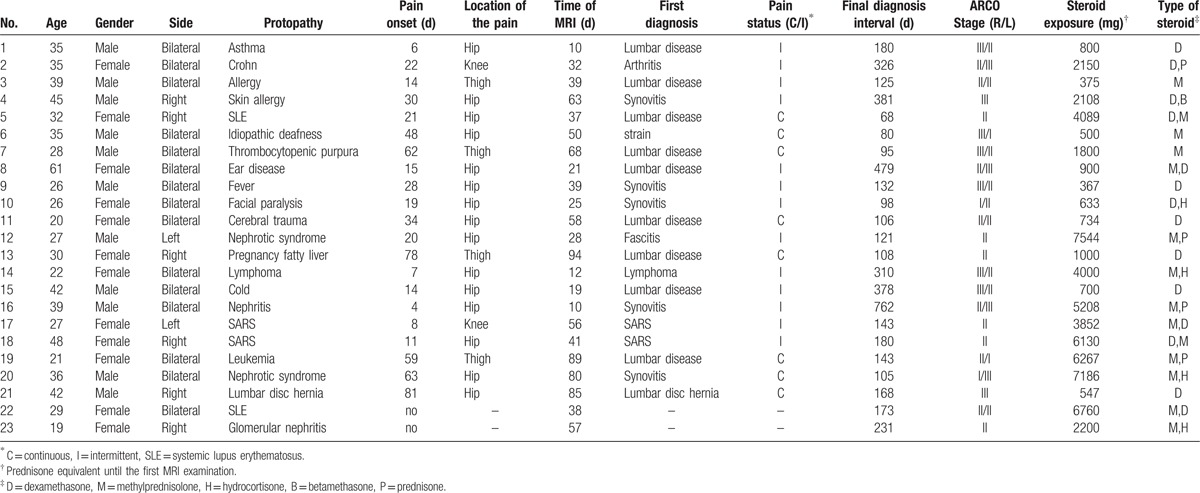
Clinical characteristics of study cohort.

In this study, 21/23 patients complained of discomfort after receiving steroid therapy but presented normal results at their first MRI examinations. Two patients did not experience any pain and underwent their first MRI examinations to screen for ONFH. Of the 21 patients who complained of discomfort, the average time from steroid administration to pain onset was 30.7 ± 24.4 days (range, 4–81 days), to the first MRI examination was 45.5 ± 26.6 days (range, 10–94 days), and to final diagnosis was 199.7 ± 173.6 days (range, 32–762 days). Time intervals were similar for male and female patients (Table 2). All 21 patients were misdiagnosed initially because the first MRI examination results were normal. One patient received a lumbar discectomy.

**Table 2:**
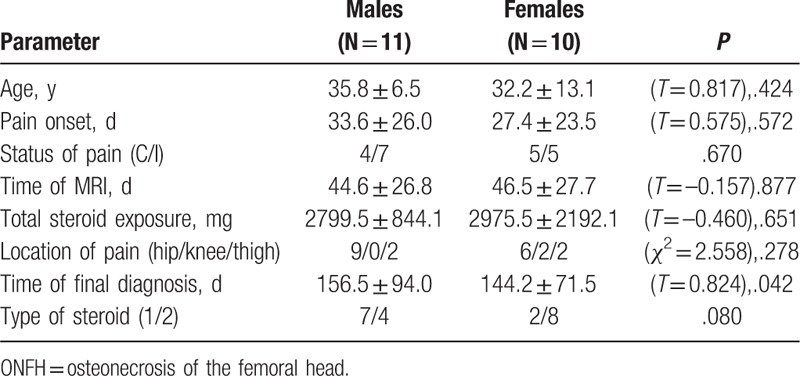
Comparison of ONFH case features between men and women.

Among the 21 patients who complained of pain, 9 patients had continuous pain, and 12 patients had intermittent pain. Pain presented relatively later in the continuous pain cases, although these patients were diagnosed at an earlier time point than the intermittent pain cases (Table 3).

**Table 3:**
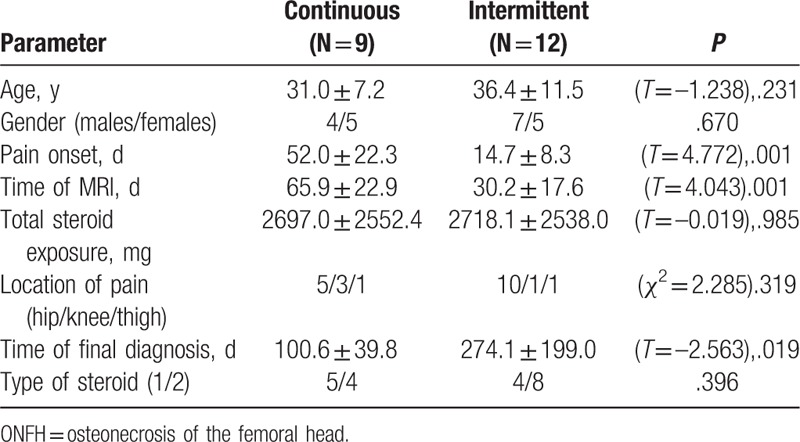
Comparison of ONFH case features between patients with continuous versus intermittent pain.

## 4 Discussion

Corticosteroid use is a leading cause of ONFH.^[10,11,15]^ However, there is a delay between the administration of corticosteroids and our ability to detect ONFH via MRI. Oinuma et al^[12]^ reported that osteonecrosis was detected in 44% of systemic lupus erythematosus (SLE) patients (32/72) by MRI approximately 3.1 months after high-dose glucocorticoid treatment. Similarly, Saito et al^[13]^ found that 48/286 patients presented with MRI evidence of ONFH within 12 months of initiation of steroid therapy following kidney transplantation. In addition, 29/34 patients showed a low-intensity band on T1-weighted images 6 to 12 weeks after renal transplantation, and 35/38 patients showed band patterns 24 weeks after renal transplantation, but none of these patients showed abnormal results 4 weeks after kidney transplantation.

Among 539 patients who received steroid therapy for SARS infection, osteonecrosis was detected within 6 months after steroid administration. All patients were rescreened by MRI 4 years later, which revealed no new lesions.^[17]^ For ONFH patients requiring continuous steroid use for treatment of background diseases, no expansion of the necrotic lesion was found.^[18]^ In a prospective study of 291 joints in 106 SLE patients without osteonecrosis after initial steroid therapy, only 6 joints developed new osteonecrosis, which only occurred after SLE recurrence in association with increased steroid doses. New lesions were delayed for a mean of 5.9 years after initial steroid administration. Mean time from SLE recurrence to appearance of new lesions was 6.2 months.^[19]^

Our results are consistent with those of previous studies. In this study, average time between steroid administration and initial MRI scan was 45.7 ± 25.5 days (range, 10–94 days) and between steroid administration and ONFH diagnosis was 199.9 ± 165.8 days (range, 32–762 days). All of these studies indicated that the adverse effects of steroid-induced osteonecrosis are time limited. Onset of ONFH might be assumed to occur 0 to 6 months after steroid use, with presentation on MRI 1 to 6 months after steroid therapy.

ONFH is an intractable disease that affects relatively young, active patients, with a mean age of 36 to 38 years.^[2,3,11]^ Most asymptomatic ONFH cases will develop to symptomatic ONFH within 5 years. Untreated ONFH has a poor outcome and often leads to subchondral collapse within a short period.^[20,21]^ Surgical head-preserving procedures are helpful to relieve pain and improve function of the affected femoral heads only in the early stage.^[4–9]^ Most patients must undergo total hip arthroplasty when the femoral head collapsed. Even with great progress in the design and technology of prostheses, revision (with unfavorable prognosis) is needed 20 to 30 years later.^[22,23]^ Thus, early diagnosis is very important for a good prognosis. Knowledge of the time lag between steroid use and MRI-detectable disease can aid us in making a detailed and reasonable screening plan. In this study, 21 cases with discomfort were misdiagnosed because the initial MRI presentation was normal. Twelve hips progressed to the collapsed stage, and 1 received lumbar discectomy after the final diagnosis. All of these events could be avoided if another MRI were performed 6 to 12 months after steroid therapy.

Characteristic presentation of ONFH is a low signal band on T1-weighted image and a high signal band on the corresponding STIR sequence. The latter corresponds to the development and calcification of vascular connective tissue between healthy and necrotic bone. Although granulation tissue development is a physiological response that contains the lesion and promotes healing, the subsequently formed fibrosis and sclerotic bone act as a barrier to revascularization of the bone marrow and the transport of the cytokines and other cellular mediators needed to remodel the bone. Necrotic bone has poor biomechanical properties. When mechanical constraints exceed bone resistance, stress fracture occurs, resulting in femoral head collapse and hip osteoarthritis. The use of some drugs, such as lipid-lowering drugs, anticoagulants, vasodilators, and traditional Chinese medicines, during the time lag period may reduce occurrence of necrosis.^[24,25]^ Other methods, such as electromagnetic stimulation, extracorporeal shock-wave therapy, hyperbaric oxygen, and core decompression, may alter the prognosis of ONFH.^[7–9]^

Previous studies^[26,27]^ reported that some patients experienced pain, mostly involving the hip and knee joints, early after treatment with steroids. In general, this “fleeting joint pain” is severe, lasts up to a week, and is accompanied by negative imaging results. Fleeting joint pain may resolve by itself or be treated with calcium antagonists. The mechanism of fleeting joint pain has yet to be elucidated but may be related to corticosteroid impulse therapy. In this study, 12/21 patients complained of short-term pain 4 to 30 days (average 14.7 ± 8.3 days) after steroid therapy. Another MRI was performed when the pain recurred. These patients met the criteria of fleeting joint pain syndrome. The remaining 9 patients complained of pain later, 52.0 ± 22.3 days after steroid therapy. This pain persisted until osteonecrosis was confirmed by a second MRI examination.

**Figure 1:**
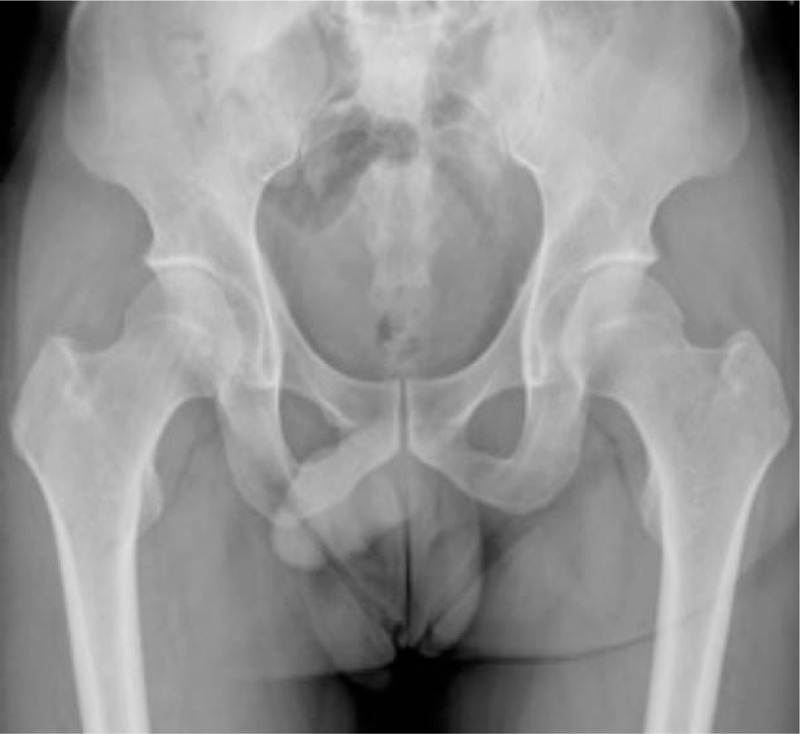
Anteroposterior radiograph of bilateral hips showing normal bony structure 48 days after corticosteroid therapy for idiopathic deafness.

**Figure 2:**
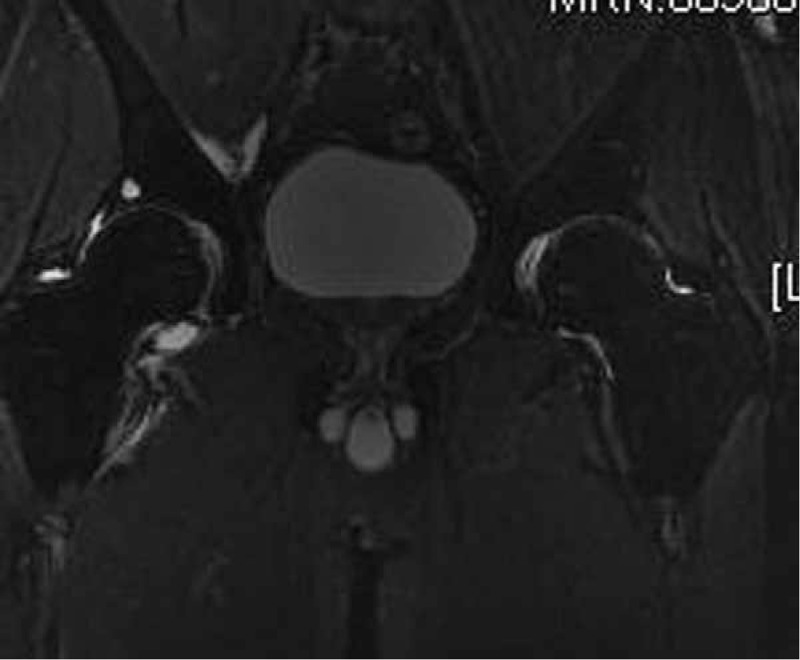
Coronal STIR image performed 50 days after corticosteroid therapy showing normal signals in both femoral heads. STIR = short τ inversion recovery.

**Figure 3:**
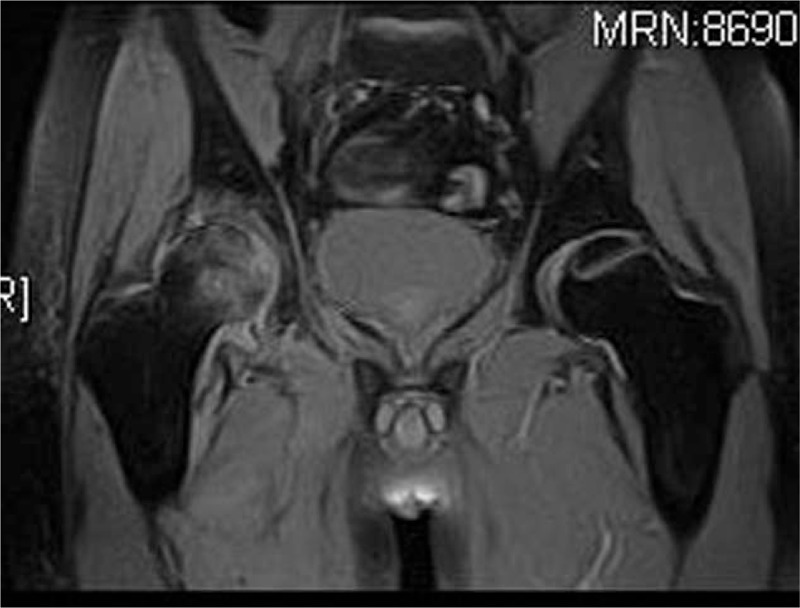
Coronal STIR image performed 80 days after corticosteroid therapy showing a high signal band with bone marrow edema in the right femoral head and an obscure high signal band in the left femoral head. STIR = short τ inversion recovery.

Case 6 complained of right hip pain 48 days after steroid administration for idiopathic deafness. X-ray and MRI performed 2 days later were normal (Figs. 1 and 2). The second MRI (80 days after steroid therapy) demonstrated features of ONFH. The contour profile of the right hip was deformed, and there was bone marrow edema (Fig. 3), suggesting that the osteonecrosis had occurred when the pain began and had weakened the mechanical structure of the femoral head. The proliferation response in the femoral head in this case was relatively weak, and the sclerosis band was not clear, consistent with a destructive repair mode and rapid disease development.^[28]^

There are several limitations in this study. Firstly, the number of cases in this research was small. We only collected 23 cases from June 2005 to December 2013. One reason for this small sample size was that most cases of early-stage ONFH were painless. Another reason was that MRI was not performed in a timely manner when patients complained of discomfort. Secondly, the nature of this study was retrospective, and MRI was performed irregularly. Large-scale prospective studies are required to study the false-negative phase of MRI after taking corticosteroids and to investigate the relationship between dosage of steroid and the presence of ONFH on MRI.
